# Targeting cistrome and dysregulated transcriptome of post-MPN sAML

**DOI:** 10.18632/oncotarget.21752

**Published:** 2017-10-11

**Authors:** Srdan Verstovsek, Warren Fiskus, Taghi Manshouri, Kapil N. Bhalla

**Affiliations:** Kapil N. Bhalla: Department of Leukemia, The University of Texas M.D. Anderson Cancer Center, Houston, TX, USA

**Keywords:** sAML, ruxolitinib, BETi, BETP-PROTAC, JAKi-persister/resistant

**Figure 1:**
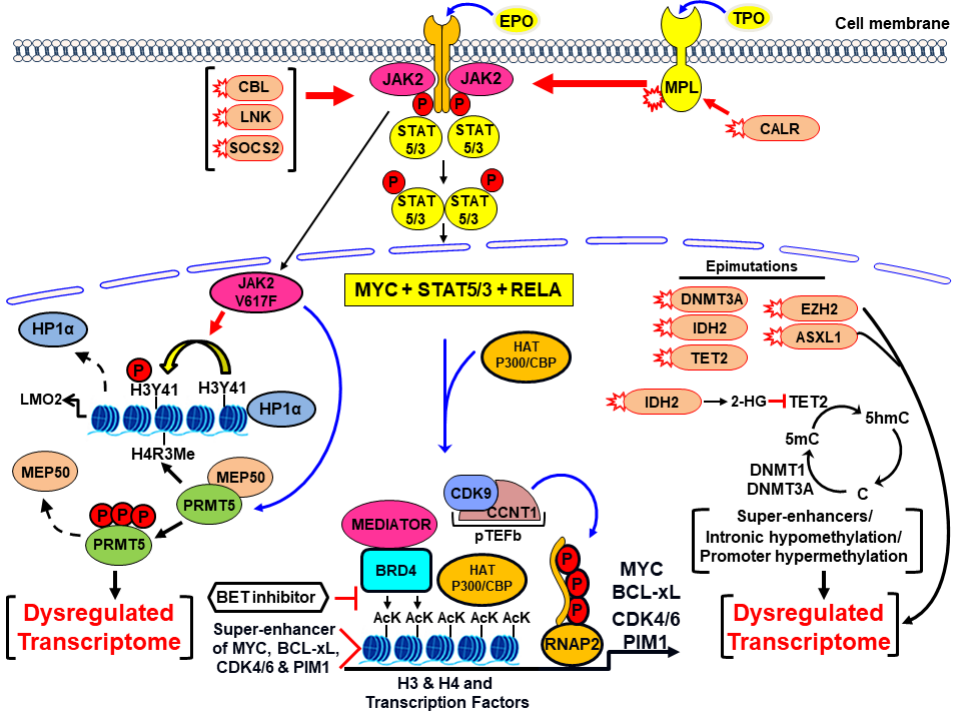
Genetic and epigenetic alterations in sAML create a dysregulated transcriptome susceptible to BET inhibitor therapy Mutations in JAK2, MPL, CALR, CBL, as well as less common genetic alterations in SOCS2 and LNK phosphorylate and increase activity of JAK2/STAT5/3. Along with MYC and other transcription factors, STAT5/3 recruits HATs and BETP to super-enhancers of growth and survival promoting oncogenes, e.g., MYC, BCL-xL, PIM1 and CDK4/6 and induces their transcript elongation through RNAP2. The epimutations in TET2, IDH2, DNMT3A, EZH2 and SRSF2 also impact the cytosine and histone methylation at enhancers and promoters, contributing to the dysregulated transcriptome. Activated JAK2 in the nucleus phosphorylates PRMT5, thereby disrupting its binding to MEP50, inhibiting symmetric arginine methyltransferase activity and de-repressing transcription. Nuclear JAK2 also tyrosine-phosphorylates H3K41, disrupting the binding of HP1α to chromatin, again de-repressing transcription. Collectively, the resulting dysregulated transcriptome is responsible for the aggressive phenotype and treatment-refractoriness of post-MPN sAML.

Pathogenic mutations in JAK2, MPL or calreticulin associated with activated JAK/STAT3/5 signaling are a common feature in Myeloproliferative Neoplasms (MPNs) [1]. Pivotal clinical trials confirmed the activity, leading to FDA approval, of the type I JAK1/2 inhibitor ruxolitinib as therapy for advanced MPN-Myelofibrosis (MF) and Polycythemia Vera [1]. Ruxolitinib confers notable clinical benefit and may improve patient survival in MPN-MF. However, adverse side effects, persistence of JAK2/STAT3/5 signaling with reduced responsiveness, and progression to secondary (s) AML are associated with loss of clinical utility of ruxolitinib [1]. While lacking additional mutations in JAK2, JAKi-persister/resistant MPN-MF or sAML cells exhibit reactivation of JAK2-STAT3/5 signaling due to trans-phosphorylation of JAK2 by JAK1 or TYK2 tyrosine kinases [2]. JAKi-persister/resistant cells exhibit dependency on heat shock protein 90 (HSP90) chaperone complex and collateral sensitivity to HSP90 inhibitor [2]. Co-mutations in TP53, ASXL1, TET2, IDH2 and SRSF2 are associated with poorer outcome in ruxolitinib-treated MF-MPN and with higher risk of AML transformation (sAML), which develops in up to 20% of patients with MPN-MF [3]. Ruxolitinib or standard anthracycline/Ara-C-based chemotherapy is only modestly active and displays limited impact on clinical outcome in sAML, which has a median survival of less than 6 months [3]. Therefore, alternative and more effective treatments are needed for sAML. It should be noted that heightened JAK2/STAT3/5 signaling and co-occurrence of mutations in epigenetic modifiers, i.e., ‘epimutations’, could potentially create the dysregulated transcriptome responsible for the aggressive phenotype and treatment-refractoriness of sAML (Figure 1) [4]. What is the underlying molecular basis of the dysregulated transcriptome in post-MPN sAML, and how could this be therapeutically approached?

Activation of multiple transcription factors (TFs), including lineage specific master regulators, MYC and signaling TFs such as STAT3/5 and RELA, and their collaborative binding to enhancers and promoters leads to recruitment of transcriptional cofactors including HATs (histone acetyltransferases) [4, 5] (Figure 1). HAT such as CBP/p300 induces acetylation of lysine residues on the histone H3 and H4 proteins and TFs [4, 5]. BRD4 and BRD2 are members of the bromodomain extraterminal (BET) family of reader proteins (BETP) that recognize and bind to acetylated lysines on histone proteins and TFs [5]. BETPs assemble transcriptional co-factors, including mediator protein and pTEFb (positive transcript elongation factor b), at super-enhancers/enhancers and promoters, thereby inducing RNA pol II (RNAP2)-mediated mRNA transcript elongation, especially of super-enhancer-driven oncogenes, including c-Myc, Cyclin D1, Bcl-xL, PIM1 and CDK4/6 that are important for cell growth and survival of AML cells, including post-MPN sAML cells (Figure 1) [4, 5]. BRD4 has also been shown to bind acetylated RELA and be essential for NFκB transcriptional activity, which is involved in the cytokine production and biology of post-MPN sAML cells [1, 5]. Several structure/activity-based BETP small-molecule, acetyl-lysine-mimetic BETP inhibitors (BETis) have been developed, including JQ1, OTX-015, GSK525762 and ABBV-075 [5]. These agents displace or evict BETPs, along with the associated transcript-initiation and elongation factors from the chromatin, causing transcriptional repression of super-enhancer-driven oncogenes [4, 5]. BETi, e.g., JQ1, but not its inactive enantiomer R-JQ1, inhibits in vitro and in vivo growth and induces apoptosis of cultured and patient-derived (PD) sAML cells, especially those expressing JAK2 V617F and mtTP53 [6]. BETi treatment repressed protein expression of c-Myc, p-STAT5, Bcl-xL, CDK4/6, PIM1 and IL7 receptor, while concomitantly inducing expression of HEXIM1, p21 and BIM in sAML cells [6]. Clinical safety and efficacy of several BETis is currently under investigation. Pre-clinically, co-treatment with BETi and ruxolitinib was shown to be synergistically active against JAKi-sensitive, whereas BETi and HSP90 inhibitor against JAKi-persister/resistant sAML cells [6]. Further pre-clinical in vivo evaluation followed by clinical testing of BETi-based combinations is warranted.

Despite exhibiting AML activity, BETi treatment leads to BRD4 protein accumulation over time [7]. Together with the reversible nature of BETi binding to BETPs, induction of BRD4/BRD2 levels by BETi treatment may account for sub-optimal BETi-mediated transcriptional repression of target oncoproteins and BETi-induced apoptosis [7]. Resistance to BETi in sAML may also be due to BETP-independent mechanism, e.g., de-repression of MYC despite BETi treatment due to an increase in nuclear localization of β-catenin and TCF4-mediated c-Myc induction [8]. To circumvent these limitations, hetero-bifunctional PROTACs (proteolysis-targeting chimeras) with two recruiting ligands connected via a linker have been designed. These include BETP-PROTAC, ARV-825 and ARV-771 (Arvinas, Inc), dBET1 and dBET6 [7]. In ARV-825, one ligand is the BETP-binding moiety (OTX015) while the other moiety (pomalidomide) specifically hijacks and recruits the E3 ubiquitin ligase Cereblon to polyubiquitylate and proteasomally degrade BETPs [7]. Unlike BETis, PROTACs can facilitate multiple rounds of sub-stoichiometric catalysis and BETP degradation. ARV-825 and ARV-771 treatment demonstrated lasting depletion of BETPs, as well as induced profound lethality in JAKi-sensitive and JAKi-persister/resistant post-MPN sAML cells [7]. Molecular basis of this activity may be explained by BETP-PROTAC-mediated attenuation of the levels of sAML-relevant pro-growth and pro-survival oncoproteins, e.g., c-Myc, Bcl-xL, CDK4/6 and PIM1, as well as inhibition of STAT5 and NFκB target gene-expressions [7]. BETP-PROTAC treatment also induces growth-inhibitory and pro-death proteins p21, p27, NOXA and BIM. ARV-771 is pharmacologically superior to ARV-825 and exerts potent in vivo anti-sAML activity in xenograft models of sAML cells [7]. BETP-PROTAC-based combinations with ruxolitinib, or ABT-263 that inhibits anti-apoptotic BCL2/Bcl-xL, or with β-catenin antagonist also need to be pre-clinically tested against JAKi-sensitive and JAKi-persister/resistant cultured and PD, post-MPN sAML cells. It would also be important to concomitantly determine genetic and molecular biomarkers that correlate with the synergistic activity of BETP-PROTAC-based combinations against sAML. Mutations in enzymes such as DNMT3A, TET2 and IDH2 found in sAML alter DNA methylation patterns, either manifesting as promoter hypermethylation or hypomethylation of distal regulatory or intronic DNA elements [4]. This could be contributing to the dysregulated cistrome and transcriptome, potentially responsible for the aggressive phenotype and treatment-refractoriness in sAML [4]. Based on this, outcome of the ongoing clinical trials of hypomethylating agents with or without ruxolitinib in genetically-profiled post-MPN sAML would also be clearly informative.
